# Characterization of System Status Signals for Multivariate Time Series Discretization Based on Frequency and Amplitude Variation

**DOI:** 10.3390/s18010154

**Published:** 2018-01-08

**Authors:** Woonsang Baek, Sujeong Baek, Duck Young Kim

**Affiliations:** Department of System Design and Control Engineering, Ulsan National Institute of Science and Technology, UNIST-gil 50, Ulsan 44919, Korea; wsbaek@unist.ac.kr (W.B.); sjbaek@unist.ac.kr (S.B.)

**Keywords:** fault detection, sensor data, frequency domain

## Abstract

Many fault detection methods have been proposed for monitoring the health of various industrial systems. Characterizing the monitored signals is a prerequisite for selecting an appropriate detection method. However, fault detection methods tend to be decided with user’s subjective knowledge or their familiarity with the method, rather than following a predefined selection rule. This study investigates the performance sensitivity of two detection methods, with respect to status signal characteristics of given systems: abrupt variance, characteristic indicator, discernable frequency, and discernable index. Relation between key characteristics indicators from four different real-world systems and the performance of two fault detection methods using pattern recognition are evaluated.

## 1. Introduction

Fault detection is a key status monitoring function that identifies the presence of faults in a system, and the times at which they occurred. Fault detection methods use pattern recognition with large datasets have been applied to various engineering areas [1] since these do not require any additional empirical knowledge to model a given system [2]. However, it is still not easy to extract meaningful patterns directly from the original datasets. For example, Hellerstein, Koutsoupias [3] demonstrated that the performance of several non-trivial pattern extraction and indexing algorithms degraded as the size of the given dataset increased. Therefore, data discretization methods have been used as a preprocessing step to reduce the dimensionality of the original dataset, while preserving the important information for pattern extraction [4,5,6]. These discretization methods commonly involve dividing the datasets into finite segments and converting each segment into an appropriate label [7]. Unsupervised discretization methods do not use the data to determine segment boundaries. Equal width discretization and equal frequency discretization are two typical unsupervised discretization methods that create continuous-valued attributes by creating a specified number of bins [8,9]. In contrast, supervised methods discretize data by considering the relations between the data values and the class information of the system. Entropy-based discretization methods is the typical supervised method, and it measures the purity of the information to determine boundaries that decrease the entropy at each interval. Maximum entropy [10] and minimum entropy [11] methods use the entropy of the information to determine suitable stopping criteria.

**Figure 1:**
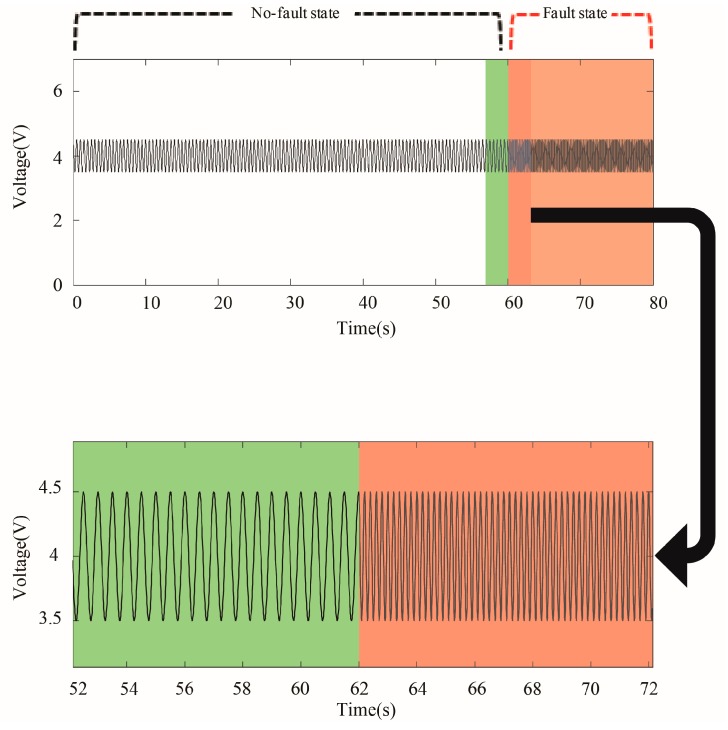
Time series where the frequency changes when the state of the system changes.

One issue with these methods is that the features that are extracted from the time domain are not always enough to distinguish between the no-fault and the fault states. For example, the amplitude of the time series shown in Figure 1 is the same throughout even though it includes both the no-fault state and the fault state. If the features that use the amplitude of the time series are identical in both states, it is difficult to discriminate between two states. Instead, the data show that the two states have different frequencies. 

Such characteristics appear in the acoustic and vibrational data that are usually collected from mechanical systems, such as rotors and bearings [12,13]. If a mechanical system has faults, then they usually generate a series of impact vibrations, and these appear with different frequencies [14]. Signal processing methods have been applied to such time series to measure and analyze the vibration responses, with the aim of detecting faults in the frequency domain [15,16,17]. Fast Fourier transforms and various of Time-frequency analysis methods are the representative signal processing methods for analyzing the frequency domain [18].

Several model-based fault detection methods using frequency features have been proposed. The performance of model-based fault detection, by nature, is dependent on a proposed system model itself. However, the operational outputs of many industrial systems are often different from the intended behaviors due to unknown disturbances, system degradation, and measurement noises [19]. Hence, observer-based approaches have been devised to combine a system model with residuals [20]. Recently, fault observer fuzzy models for complex nonlinear dynamic processes have been presented in finite-frequency domain [21,22,23]. 

Data-driven approaches have been develop to make fault status decisions by analyzing a large amount of historic data in the case of absence of any predefined system model [24]. Many studies have been made to discover fault patterns, i.e., particular time-frequency features, via various machine learning techniques [25,26,27,28]. Here, it is necessary to compose system status patterns by using either amplitude variation in time domain or frequency variation in frequency domain. 

Therefore, the first objective of this study is to develop a pattern extraction method that discretizes the frequency components of multivariate data. In Section 2, the labels are defined using features in the frequency domain. The procedures for discretization and pattern extraction follow those of previous work that used time-domain features to construct labels [29].

The second objective of this study is to provide a guideline for selecting appropriate labels for fault pattern extraction via a comparison between the performance of different pattern extraction methods and the representative characteristics that they extract from the time series. In the process, several key characteristics indicators (KCIs) and an aggregated characteristic indicator is suggested. The guideline is also proposed regarding the type of labels that should be selected based on the key characteristics. The key characteristics of the time series and the experimental study are introduced in Section 3 and Section 4. The nomenclature regarding indices, parameters, and variables is summarized in Table 1.

## 2. Methodology

### 2.1. Frequency Variation-Based Discretized Time Series Generation

Conventional discretization methods downsize time series into a finite number of bins that preserve the temporal information of the original time series. Such methods have been applied to fault detection, owing to their efficiency in extracting patterns using discretized labels that are derived from statistical features of the discretized bins [5]. However, these methods assume that the statistically discernible features needed for fault detection exist in the time domain. In the case of vibrational systems, where the dominant features generally lie in the frequency domain; this means that the patterns for fault states and no-fault states cannot be distinguished. Therefore, we propose a new discretization method that uses frequency domain features as labels, following the systematic discretization method introduced in previous work [29]. The method is briefly described in the following section.

### 2.2. Discretization-Based Fault Pattern Extraction

The discretization-based fault pattern extraction method aims to extract a set of fault patterns from the training data of a given system for fault detection. Therefore, the information of no-fault state and fault state, namely fault time markers, are assumed to be given a priori. The method comprises three steps: label definition, label specification, and fault pattern extraction.

The label definition step involves estimating the distribution model of the time series, and dividing the given time series into a finite number of bins. Assume that a given time series is collected from a system containing I sensors. Let $X_{i}=\left[x_{i1},\ldots ,x_{ij},\ldots ,x_{iJ}\right]$ be the J data points in the time series gathered by the *i*th sensor and $X=\left[X_{1},\ldots ,X_{i},\ldots ,X_{I}\right]$ be a set of sensor data.

Then, the probability density function (PDF) is estimated to determine the distribution model of the given data set. The probability density function, $PDF_{opt}$, whose statistical characteristics are most similar to those of the dataset is determined by computing the optimal likelihood values between the histogram of the original datasets and the PDF candidates.

**Figure 2:**
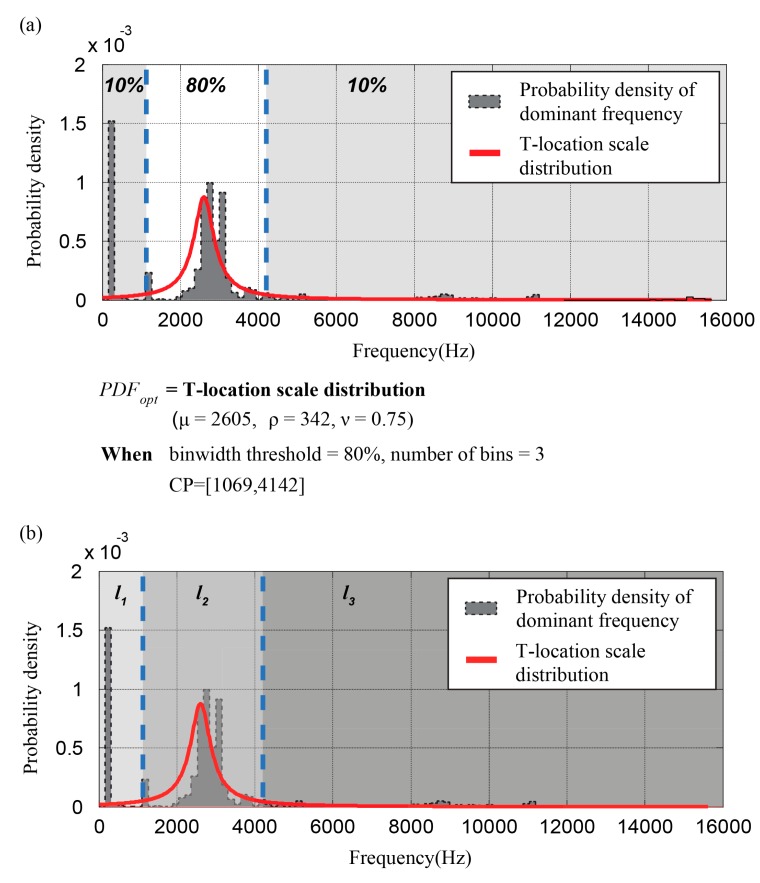
An example of probability density function (PDF) estimation for a dominant frequency of microphone sensor data collected by the automotive buzz, squeak, and rattle (BSR) noise detection system. (**a**) $PDF_{opt}$ of the sensor data, which follows a T-location scale distribution. The cut points of the dominant frequency, [1069 Hz, 4142 Hz] is derived with the user-defined discretization parameters, bin width, and the number of bins; (**b**) A set of labels [$l_{1}\;l_{2}\;l_{3}]$ is determined according to the number of cut points and the feature of each bin.

After that, a set of cut-points $\mathrm{CP}\left(X_{i}\right)$ are generated using the set of segments, $PDF_{opt}$, and the discretization parameters b, which determines the number of bins, and bw, which defines the size of the central bin that contains the centroid of $PDF_{opt}$. We used odd values for b to generate balanced sets of labels where the probability density of each bin, except the center bin, was identical. After setting these parameters, a set of cut-points is derived from the data from each sensor, $\mathrm{CP}\left(X_{i}\right)=\left[CP_{i1}\;CP_{i2}\ldots CP_{i\left(b-1\right)}\right]$, followed by a set of labels $L\left(X_{i}\right)=\left[l_{i1}\;l_{i2}\ldots l_{ib}\right]$. For instance, Figure 2 shows a procedure of the label definition step.

The label specification step divides the data into s discrete segments and attaches the corresponding labels to them. A matrix consists of labeled segments are named as the discrete state vector, $D\left(X_{i}\right)=\left[d_{i1},\cdots ,d_{is},\cdots ,d_{iS}\right]$ where the $d_{is}$ represents the label assigned to the *s*th segment. Here, the label for each segment is determined by its relative location of the average value of the amplitude. Then, X is converted into a set of discrete state vector, D(X):

(1) $$D\left(X\right)=\left[\begin{array}{c} D(X_{1})\\ \vdots \\ D(X_{i})\\ \vdots \\ D(X_{I}) \end{array}\right]=\left[\begin{array}{ccccc} d_{11} & \cdots & d_{1s} & \cdots & d_{1S}\\ \vdots & \ddots & \vdots & \ddots & \vdots \\ d_{i1} & \cdots & d_{is} & \cdots & d_{iS}\\ \vdots & \ddots & \vdots & \ddots & \vdots \\ d_{I1} & \cdots & d_{Is} & \cdots & d_{IS} \end{array}\right]$$

**Figure 3:**
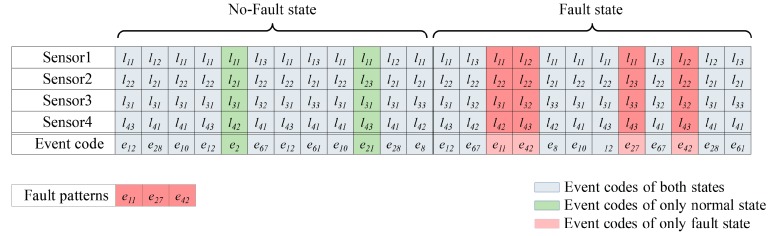
An example of the fault pattern extraction procedure (dataset with four sensors): Each sensor data (Sensor 1, Sensor 2, Sensor 3, Sensor 4) has same length, and discretized into 24 segments. Meanwhile, a set of cut-points of each sensor data are derived by PDF estimation, followed by a set of labels $L\left(X_{i}\right)=\left[l_{i1}\;l_{i2}\;l_{i3}\right]$. Then, designated labels in each column of four sensor data are converted into unique event codes, which are depicted as $E=\left[e_{1}\;e_{2}\ldots e_{81}\right]$. As a result, $e_{11},e_{27},e_{42}$ are selected as fault patterns.

To represent the state of the system in terms of the labels that are assigned to each sensor, *m* labels for a given time index, i.e., a column vector of **D**(**X**), are converted into event codes. The event codes are combination of all the possible discrete state vectors, where a total number is $b^{I}$. The event codes are assigned into the set of discrete state vectors, which the composition of discrete state vectors is same. For instance, in Figure 3, we defined the first event code, $e_{1}$ as [$l_{11}\;l_{11}\;l_{11}\;l_{11}$], the second event code, $e_{2}$ as [$l_{11}\;l_{11}\;l_{11}\;l_{12}$], and so on. We assigned $e_{2}$ to the fifth set of discrete state vectors due to the same composition of discrete state vectors. 

The set of fault patterns $F_{p}$ is composed of the event codes that only occur in fault states of the system, as Figure 3 shows. In other words, $F_{p}$ is the relative complement of $P_{f}$ in $P_{n}$, where $P_{f}$ is the set of distinct event codes that occur in fault states and $P_{n}$ is the set of distinct event codes that occur in no-fault states. The method then determines the state of the given time series **X** depending on whether there is an element of $F_{p}$ in **D**(**X**).

As performance of the fault detection is determined as follows: the number of the fault states where a specific fault pattern is found. Herein, the label definitions play a key role in this scheme. In the next section, we propose a new label definition using the frequency-domain features to expand the coverage of the discretization method.

### 2.3. Dominant Frequency Extraction for Each Segment

**Figure 4:**
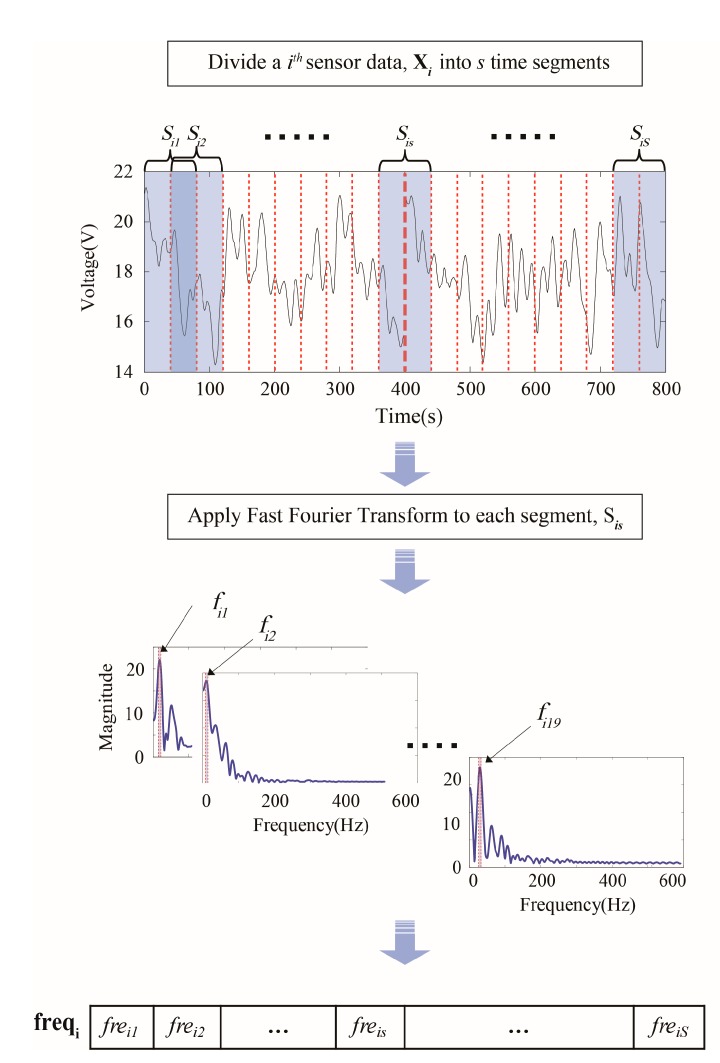
Extraction of the dominant frequency in the time series.

Frequency components have been popularly used to fault detection, Fast Fourier transforms is one of methods to extract frequency components from the original sensor signals of the system [26]. However, in general, traditional FFTs require periodic and stationary datasets, and are not directly applicable to analyze the time dependent frequency variation of sensor signals, whereas many datasets of interests exhibit non-stationary characteristics [30]. Time-frequency analysis methods, such as short time Fourier transforms (STFTs) and wavelet transforms (WTs), have been suggested to analyze non-stationary signals by applying FFT to the segmented signals in each time window [18]. Therefore, we applied STFT in order to combine a time-frequency analysis into temporal discretization methods for label definition. Figure 4 shows an example of dominant frequency extraction for label definition in the frequency variation-based discretization procedure.

Before defining the labels, the time series $X_{i}$ is divided into segments using the predetermined parameter, w, which represents the size of the segment. The number of segments S is therefore equivalent to J/w, where J is the number of data points in $X_{i}$. If the segmentation process leaves a remainder, this is ignored on the basis that it does not contain a significant amount of information. For segmentation, we applied the sliding window method to preserve sequential information [31]. The sliding window overlap was set to half of the window size w. The number of segments S is therefore equivalent to 2J/w−1. Let $X_{i}$ be segmented into the total S segments, and then for a given window size w and sliding window size w/2, the set of data points in the *s*th time segment of the *i*th sensor, $S_{\mathrm{is}}$ is given by:

(2) $$S_{\mathrm{is}}=\left\{x_{\lceil \frac{w}{2}\rceil \times \left(s-1\right)+1},x_{\lceil \frac{w}{2}\rceil \times \left(s-1\right)+2},\;\ldots \;,\;x_{\lceil \frac{w}{2}\rceil \times \left(s-1\right)+w}\right\}$$

The procedure is done with the window size of a time segment = 40 s, the sampling rate = 1 kHz, *s* = 19. $X_{i}$, which iss *i*th sensor data, is divided into s segments, and FFT is applied to each segment for extracting the dominant frequency. Finally, a sequence of dominant frequency, ${\mathrm{fre}}_{i1}$ is acquired.

We can now represent the overall sensor dataset X and the time series $X_{i}$ from each sensor in terms of these sets of segments. The following label definition is now proposed. Let $\omega_{isv}$ denote the Fourier coefficient of the vth sinusoid for the *s*th time segment signal of the *i*th sensor. We can obtain the Fourier coefficient $\omega_{isv}$ by applying a FFT to $S_{\mathrm{is}}$, where the Fourier component v=1,2,…,V.

(3) $$\omega_{isv}=\overset{w}{\underset{t=1}{\sum }}\left(x_{\lceil \frac{w}{2}\rceil \times \left(s-1\right)+t}\times e^{-\frac{2\pi j}{w}\times vt}\right)$$

Let $\omega_{\mathrm{is}}$ denote the vector, ${\left[\omega_{is1},\ldots ,\omega_{isv},\ldots \omega_{isV}\right]}^{T}$ and the Fourier matrix **W*_i_*** for the *i*th sensor data is then defined as follows.

(4) $$W_{i}=\left[\Omega_{i1},\;\cdots ,\Omega_{is},\;\cdots ,\;\Omega_{iS}\right]$$

For the sake of simplicity, without a loss of generality, we employ the dominant frequency having the highest peak in the frequency spectrum as a key feature to characterize the inherent information in the frequency domain. The dominant frequency in the *s*th segment of the *i*th sensor data is denoted as $fre_{is}.$ Finally, a vector of dominant frequencies ${\mathrm{freq}}_{i}$, is obtained from the $W_{i}$, as follows.

(5) $${\mathrm{FREQ}}_{i}=\left(fre_{i1},\ldots ,fre_{is},\ldots ,fre_{iS}\right)$$

### 2.4. Characterization of System Status Signals

The fault pattern extraction performance is highly affected by whether appropriate labels are selected to characterize the time series. To ensure that the labels are suitable, we need to determine the relation between the characteristic indicators for the collected signal and the fault pattern extraction performance. In this section, three KCIs are introduced to represent the characteristics of the time series of the given signal: abrupt variance (*aVar*) and discernibility index (*DI*) from the previous research, and discernable frequency (*DF*). Furthermore, we define *CI* as an aggregated characteristic index of *DI* and *DF*. We analyzed four datasets of multi-sensor signals and represented the main feature of each dataset by the three KCIs and the aggregated index *CI*. We discuss the relationships between fault detection performance and KCIs. 

*aVar* was devised by multiplying a square sum of differences between adjacent data points to conventional variance for determining the magnitude of the abrupt and steady changes, and it is given by following equation [29].

(6) $$aVar_{i}=\frac{\sum {\left(x_{ij}-{\bar{x}}_{i}\right)}^{2}}{J}\times \frac{\sum {\left(\left(x_{ij+1}-x_{ij}\right)-\bar{\left(x_{ij+1}-x_{ij}\right)}\right)}^{2}}{J-1}$$

In contrast, the *DI* measures the degree of statistical overlap between no-fault and fault states, as follows [29]:

(7) $$DI_{i}=\int \min\{PDF^{nf}\left(x\right),\;PDF^{f}\left(x\right)\}x$$

where $PDF^{nf}\left(x\right)$ and $PDF^{f}\left(x\right)$ represent the estimated PDFs for the sensor data *x* in the no-fault and the fault states respectively.

We further define the *DF* to represent the frequency similarity between fault and no-fault states in terms of the composition of the time-frequency components. Existing features in the frequency domain, such as the dominant frequency and power spectral density, cannot represent time-frequency characteristics of the data because they utilize a classical FFT [19]. The *DF* is therefore defined as follows.

(8) $$DF_{i}=\int \min\left\{PDF^{nf}\left(f\right),\;PDF^{f}\left(f\right)\right\}df$$

**Figure 5:**
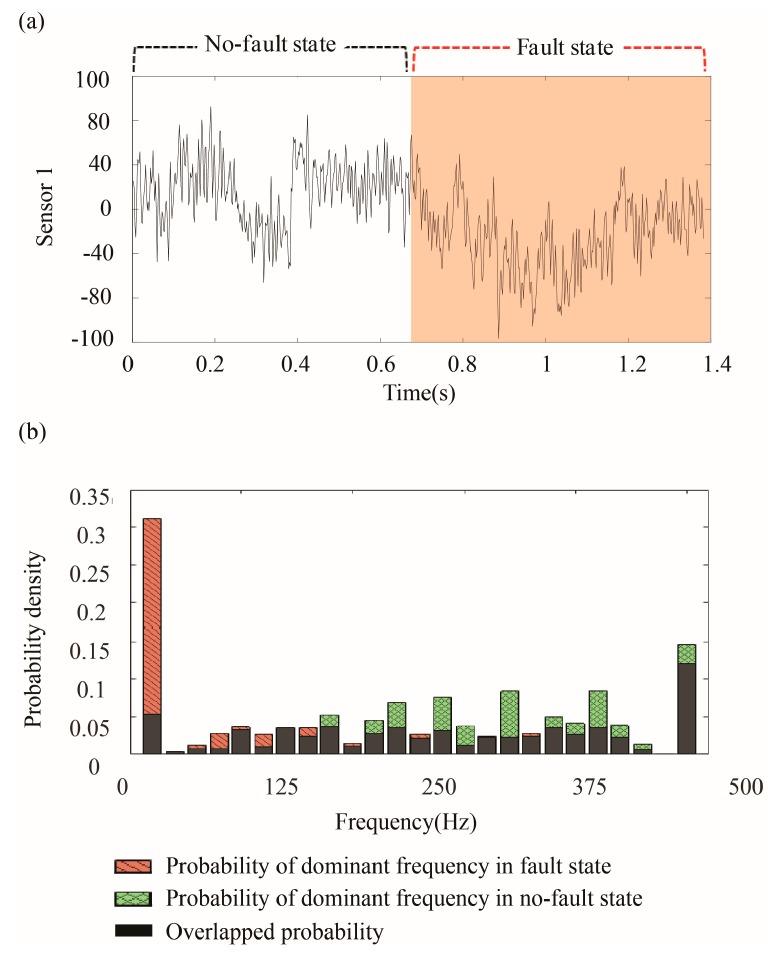
Calculation of *DF* (*DF* = 0.331): (**a**) A sensor signal; and, (**b**) the probability density of dominant frequencies in the fault, the no-fault states, and the overlapped probability density between them.

$PDF^{nf}\left(f\right)$ and $PDF^{f}\left(f\right)$ represent the estimated PDFs of the frequency of the *i*th sensor signal in the no-fault and the fault states respectively. Figure 5 shows an example *DF* calculation, giving a frequency similarity of 0.331.

The *CI* is further defined to simultaneously highlight the performance of the *DI* and *DF* for the given data set. It is calculated by multiplying the complement of the *DI* with the *DF*, as follows.

(9) $$CI=\left(1-\frac{1}{I}\overset{I}{\underset{i=1}{\sum }}DI_{i}\right)\;\times \;\frac{1}{I}\overset{I}{\underset{i=1}{\sum }}DF_{i}$$

## 3. Computational Experiment

In this section, we evaluate the performance of the discretization-based fault pattern extraction method by considering both frequency and amplitude variation, and discuss the relationship between the KCIs of system status signals and performance of fault detection. The detailed description of the experimental data is listed in Table 2.

Previous studies used a statistical representative sensor value and/or its linear trend in each time segment to construct a set of labels. We will call the fault patterns that are generated by the amplitude variation of sensor values as *FP1*, and those generated by the frequency variation of sensor values as *FP2*. The two pattern generation methods were applied to real-world datasets collected from four mechanical systems. The following subsections describe each system and provide brief information regarding the performance of pattern extraction with respect to the KCIs. The results are summarized in Table 3. 

### 3.1. Laser Welding Monitoring Data

**Figure 6:**
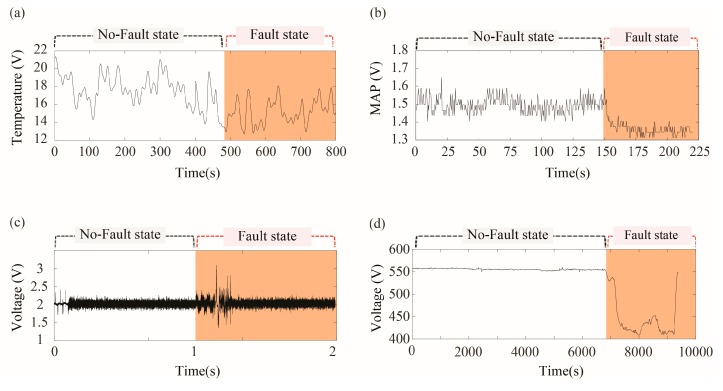
Sensor data collected from four different systems having no-fault state and fault state: (**a**) temperature signals collected from the laser welding monitoring system; (**b**) MAP (Manifold Absolute Pressure) signals collected from the vehicle diagnostics simulator; (**c**) microphone sensor signals collected from the automotive BSR noise monitoring system; and, (**d**) sensor signals for turbocharger inlet temperature collected from the marine diesel engine.

**Figure 7:**
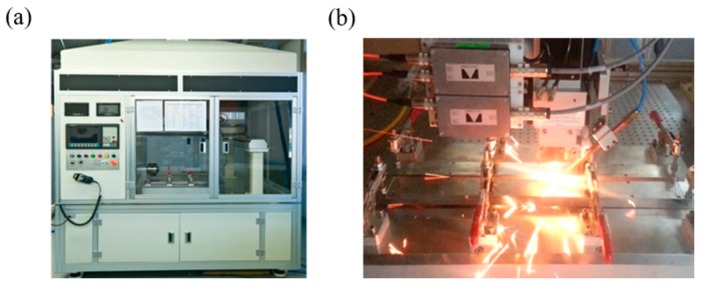
Laser welding monitoring system: (**a**) Laser welding station and (**b**) PRECITEC laser welding monitoring sensors.

Laser welding monitoring data, shown in Figure 6a, were collected from a laser welding system, as shown in Figure 7. This system was originally developed to examine the relationship between the part-to-part gap of two galvanized steel sheets and the weldment quality of a joint. The system consisted of PRECITEC LWM^TM^ (Precitec, Gaggenau, Germany) as a data acquisition device. The system used a IPG YLS 2000 AC fiber laser source (IPG Photonics, Oxford, MA, USA) with a maximum output discharge of 2 kW.

We controlled the gap between the galvanized steel sheet parts by inserting a conventional metal thickness gauge having thickness of 0.1 mm to 1.0 mm. The travelling path of the laser was defined as ascending direction. In this study, we generated five defective weldments by controlling the artificial gap (=0.5 mm) between specimens, and forty-five normal weldments without gap.

The computed KCIs are listed in Table 2, showing low *aVar* and *DI* but high *DF* and CI. By using *FP1*, we could detect all the five weld defects, while only two defects were identified by *FP2*.

### 3.2. Automotive Gasoline Engine Data

**Figure 8:**
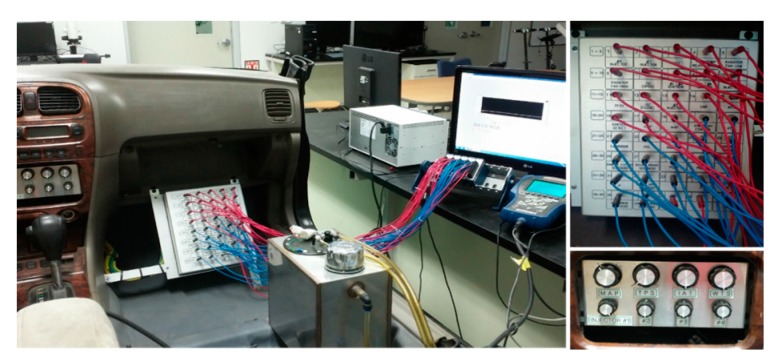
Vehicle diagnostics simulator: HYUNDAI SIRIUS-II engine, NI 9221 data acquisition device, and eight sensor voltage controllers.

Automotive gasoline engine data, shown in Figure 6b, were collected from a vehicle diagnostics simulator, shown in Figure 8. The system consists of 40 sensors on SIRIUS-II engine (Hyundai Motors, Ulsan, Korea) and NI compact DAQ system (NI 9221) as a data acquisition device. The system can simulate a fault of the engine by directly controlling an intake airflow, or the other actuators in the fuel injection system.

In this study, we artificially generated the fault that stops the engine by increasing an amount of air in the intake manifold. It was conducted with following steps:

- Step 1. Turn on the engine
- Step 2. Control the amount of manifold air flow
- Step 3. An engine knocking occurs

The data in no-fault state was collected during Step 1 and Step 2, while the data in the fault state was collected in Step 3.

The sensor signals show high *aVar*, *DI*, *DF*, and *CI*. By *FP1*, we could detect 46 engine faults, while all of the 46 faults were identified by *FP2*.

### 3.3. Automotive Buzz, Squeak, and Rattle (BSR) Noise Monitoring Data

**Figure 9:**
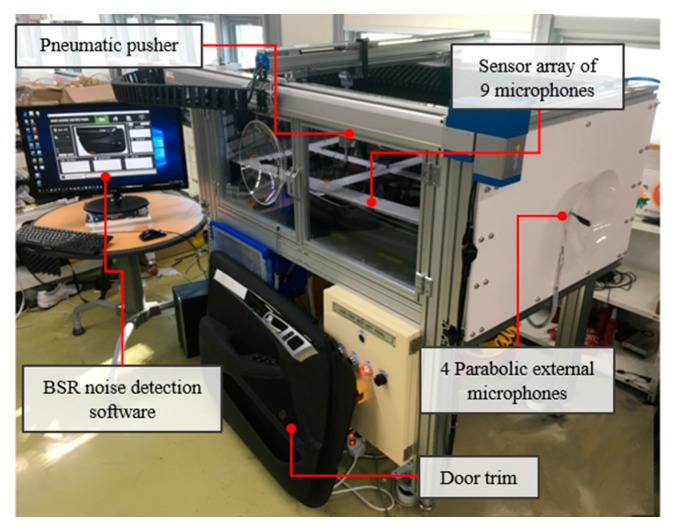
Automotive BSR noise monitoring system: a sensor array of nine microphones, four parabolic microphones, a pneumatic pusher controlled by a gantry robot, and NI cDAQ-9178 ^TM^ data acquisition device.

The BSR noise monitoring data, as shown in Figure 6c, were collected from the automotive BSR monitoring system, as shown in Figure 9. BSR noises are induced by a friction between automotive subcomponents. The system was developed to detect a defective car door trim during an assembly process that has a potential to generate BSR noises. The developed in-process BSR noise detection system consists of a sensor array of nine microphones, four parabolic microphones, a pneumatic pusher controlled by a gantry robot, a data acquisition system, NI cDAQ-9178^TM^ (National Instruments, Austin, TX, USA), and a noise detection software that we developed.

A car door trim was slowly pressed down by a pneumatic pusher with a pressure of 10 kgf/cm^2^. We then monitor the acoustic signals measured right above the door trim by a microphone array in order to identify BSR noises. We determined the state of a door trim whether it generated abnormal sounds when pressed by a pneumatic pusher.

The sensor signals show low *aVar* and *CI* but high *DI* and *DF*. By *FP1*, we could detect 80% of the fault states, while all the fault states were identified by *FP2*.

### 3.4. Marine Diesel Engine Data

Marine diesel engine data, shown in Figure 6e, were collected from a marine diesel engine (Type 9H32/30, Hyundai Heavy Industries, Ulsan, Korea). We selected six major sensors in the modularized feed system among the 482 sensors installed in the engine. We defined the abnormal combustion as the fault state of the engine. It has usually occurred when a cylinder’s temperature exceeds a predefined control limit. The sensor data shows low values for *aVar*, *DI*, *DF*, and *CI*. By *FP1*, we could detect 93% of the fault states, while all of the fault states were identified by *FP2*.

## 4. Concluding Remarks

The four different datasets were analyzed with two different discretization-based fault pattern extraction methods. The results demonstrated that the fault pattern extraction performances were closely related with the KCIs. The performance of *FP2* decreased as the *DF* and *CI* increased; in particular, the laser welding data showed the lowest performance (40%) together with the highest *DF* and *CI* values. When the *DF* is high, *FP2* cannot generate enough fault patterns due to low event code variation between the no-fault and the fault states. The performance of *FP2* also decreases as *DI* increases.

Based on these empirical studies, we can provide a guideline for selecting an appropriate label definition method in accordance with the KCIs of the given multivariate time series data. By definition, if the difference between no-fault and fault states is more distinct in the frequency domain, then the *CI* value will be relatively low. We observed that the frequency variation based discretization method produced a better result when the *CI* is low.

In summary, we have proposed a new fault pattern extraction method using frequency variation-based discretization. For fault pattern extraction, first, the time series of a sensor signal is discretized to create a set of labels representing the dominant frequency of each time segment. Second, the pattern is generated by converting series of labels into a set of event codes. Third, the fault patterns are determined as the patterns that only occur in a fault state of a system.

In addition, we have investigated a relation between the KCIs and the performance of fault pattern extraction using both amplitude and frequency variation, providing a guideline for selecting appropriate label definitions. The results show that the *CI*, the aggregated index of the *DF* and the *DI*, can be used as a good reference for selecting an appropriate label definition method. For example, the fault pattern performance of *FP2* decreased as *DF* and *CI* increased. In contrast, *aVar* was only weakly related to the *FP2* performance. Furthermore, the *FP1* performance decreased as the *DI* increased, whereas *CI* decreased. The experimental result confirmed that the frequency variation based discretization method produced better result when the *CI* is low. Since the fault pattern performance is also closely related to the discretization parameters, and therefore, further empirical study is necessary.

## Figures and Tables

**Table 1:** Nomenclature.

Notation	Description
Indices	
I	number of the sensors (*i* = 1, 2, …, *I*)
J	number of the data points (*j* = 1, 2, …, *J*)
S	number of discretized segments (*s* = 1, 2, …, *S*)
*V*	number of frequency bins (*v* = 1, 2, …, *V*)
*Z*	number of event codes (*z* = 1, 2, …, Z )
Parameters	
*b*	number of bins
bw	size of central bin
w	window size of each segment
Variables	
$X_{i}$	time series of *i^th^* sensor signal
X	discretized time series of $X_{i}$
$PDF_{opt}$	estimated probability density function of given data
$l_{ib}$	*b^th^* label defined at label definition step
$CP_{i\left(b-1\right)}$	*(b − 1)^th^* cut point defined at label definition step
$L\left(X_{i}\right)$	a set of labels for $X_{i}$: $\left[l_{i1}\;l_{i2}\ldots l_{ib}\right]$
$\mathrm{CP}\left(X_{i}\right)$	a set of cut points for $X_{i}$: $\left[CP_{i1}\;CP_{i2}\ldots CP_{i\left(b-1\right)}\right]$
$d_{is}$	*s^th^* discrete state vector of *i^th^* sensor
**D(X)**	a set of discrete state vector of X
$e_{z}$	*z^th^* event code of X, where *z* = $b^{I}$
E	a set of event codes of X
$P_{n}$	a set of event codes occurs in no-fault state
$P_{f}$	a set of event codes occurs in fault state
$F_{p}$	a set of event codes which only occur in fault state
$fre_{is}$	*s^th^* dominant frequency of *i^th^* sensor
${\mathrm{freq}}_{i}$	a set of dominant frequency of *i^th^* sensor

**Table 2:** Description of experimental datasets.

Dataset	Sensors	Total Number of Faults	Sampling Rate	Acquisition Time
laser welding	plasma intensity weld pool temperature back-reflection	5 defects among 50 weldments	1 KHz	0.4 s/specimen
gasoline engine	crank position manifold absolute pressure throttle position #1 injector position; #2 injector position; #3 injector position	50 deliberate engine knockings for 3 h engine run	2 Hz	3 h
BSR noise	sensor array of 9 microphones 4 parabolic external microphones	22 defects among 47 door trims	32,768 Hz	2 s/inspection
marine diesel engine	air cooler pressure turbocharger inlet temperature lube oil inlet temperature turbocharger speed current for ignition power factor	19 abnormal combustions for 2 months	1 Hz	2 months

**Table 3:** Fault detection performance and key characteristics indicators (KCIs) for the four datasets.

Dataset	KCIs				Fault Detection Performance	
	*CI*	*DF*	*DI*	*aVar*	*FP1* Amplitude Variation	*FP2* Frequency Variation
Laser welding monitoring	79.1	0.813	0.027	0.004	100%	40%
Automotive gasoline engine	51.5	0.671	0.232	0.034	92%	92%
Automotive BSR noise monitoring	12.0	0.522	0.770	0.004	80%	95%
Marine diesel engine	19.9	0.208	0.042	0.007	93%	100%
